# Supplementary material for: Neutron scattering and neural-network quantum molecular dynamics investigation of the vibrations of ammonia along the solid-to-liquid transition
Source: Nat Commun. 2024 May 9;15:3911. doi: 10.1038/s41467-024-48246-9 (PMC11082248; doi:10.1038/s41467-024-48246-9)
Supplement: Supplementary file 1 — Supplementary Information [file 41467_2024_48246_MOESM1_ESM.pdf]

Supplementary Information for: "Neutron scattering and neural-network quantum molecular dynamics investigation of the vibrations of ammonia along the solid-to-liquid transition"

Authors

Authors

T. M. Linker[1,2], A. Krishnamoorthy[3], L. L. Daemen[4], A. J. Ramirez-Cuesta[4], K. Nomura[1], A. Nakano[1], Y. Q. Cheng*[4], W. R. Hicks[4], A. I. Kolesnikov**[4], and P. D. Vashishta***[1]

Affiliations

1. Collaboratory for Advanced Computing and Simulations, University of Southern California, Los Angeles, CA 90089-0242, USA

2. Stanford PULSE Institute, SLAC National Accelerator Laboratory, Menlo Park, California 94025, USA

3. Department of Mechanical Engineering Texas A&M, 400 Bizzell St, College Station, TX 77843, USA

4. Neutron Scattering Division, Oak Ridge National Laboratory, Oak Ridge TN, 37831, USA

Corresponding Authors

*chengy@ornl.gov

**kolesnikovai@ornl.gov

***priyav@usc.edu

Notice: This manuscript has been authored by UT-Battelle, LLC under Contract No. DE-AC05-00OR22725 with the U.S. Department of Energy. The United States Government retains and the

publisher, by accepting the article for publication, acknowledges that the United States Government retains a non-exclusive, paid-up, irrevocable, world-wide license to publish or reproduce the published form of this manuscript, or allow others to do so, for United States Government purposes. The Department of Energy will provide public access to these results of federally sponsored research in accordance with the DOE Public Access Plan (http://energy.gov/downloads/doe-public-access-plan).

Comparison of NH_3 Vibrational Spectrum Measured with SEQUOIA and VISION Spectrometers

We report the measured integrated dynamic structure factor for 3 different incident energies on direct geometry SEQUOIA spectrometer which is illustrated in Fig. S1, a-c. For comparison we have plotted the total integrated dynamic structure factor measured by indirect geometry spectrometer VISION in Fig. S1d. The two different measurements of NH_3 dynamic structure factor are in good agreement.

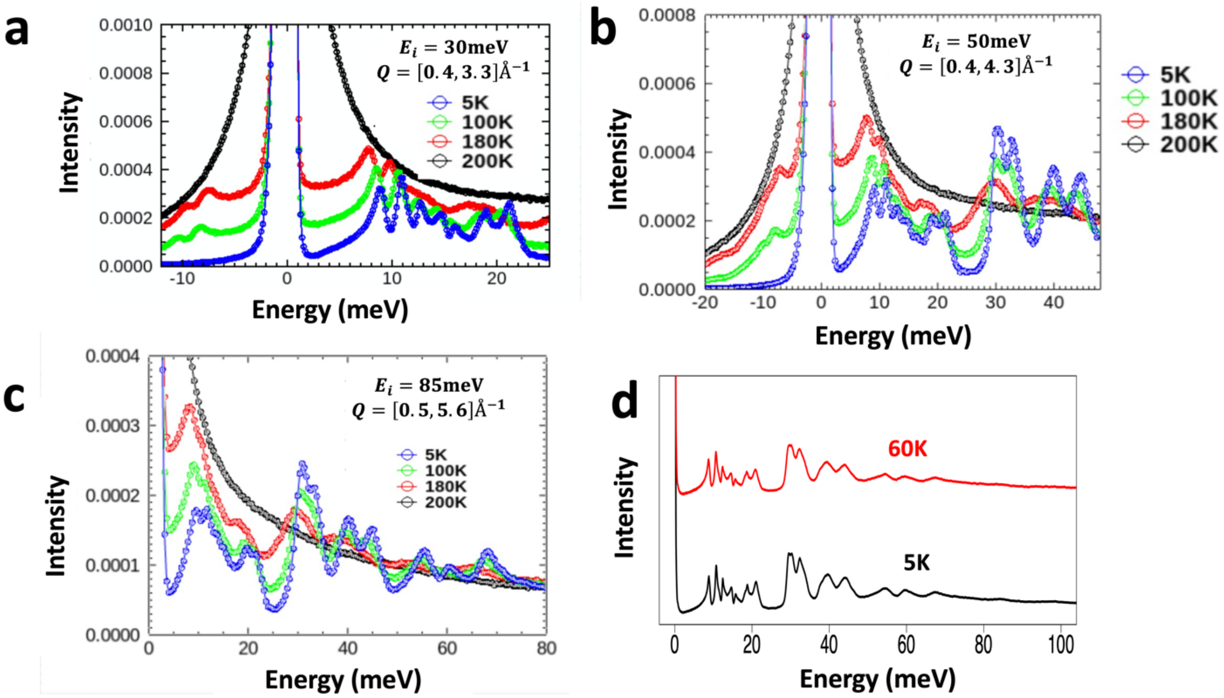

Figure S1. Comparison of SEQUOIA and VISION measurement for NH_3 integrated dynamic structure factor. (a)-(c) SEQUOIA measurement at 3 different incident energies. (d) Integrated dynamic structure factor measured by VISION spectrometer (the spectra are vertically shifted for clarity) that is in good agreement with SEQUOIA measurements.

Neutron Recoil Scattering on Ammonia

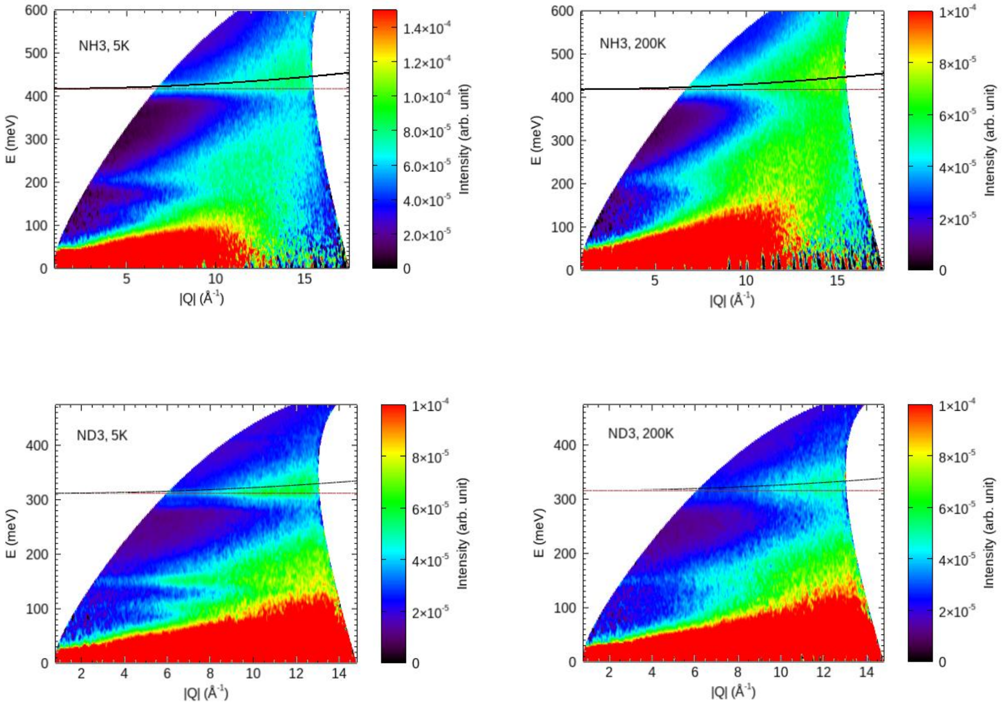

Figure S2. *S(Q,E)* spectra for NH_3 (top) and ND_3 (bottom) measured with E_i=700 and 500 meV respectively at 5 K (left) and 200 K (right). Black lines describe shift of the stretching modes due to neutron recoil scattering on free particles of mass 17 and 20 a.u. for NH_3 and ND_3 respectively, (for ND_3 the recoil line is plotted only for the antisymmetric modes); the horizontal brown lines correspond to the Gaussian fitted values for the respective modes.

The *E*–*Q* dependence for neutron recoil scattering [Tomkinson, J. The effect of recoil on the inelastic neutron scattering spectra of molecular vibrations. *Chem. Phys.* **127**, 445-449 (1988).] on a free particle of mass *M* can be described as $E_R=\hbar^2Q^2/2M$. The *Q* dependence of the N-H(D) stretching modes peaks in *S(Q,E)* spectra in Fig. S2 can be reasonably well described by neutron recoil scattering on protonated and deuterated ammonia molecules (with *M*=17 and 20 a.u., respectively), so the molecules in the solid and liquid states of ammonia are weakly bound. The *S(E)* spectra for stretching modes shown in the Fig. 2 (b, c, e, f) were obtained by summation of *S(Q,E)* over *Q* range from ~6 to 10 Å^{-1}, therefore, to get the real values for the stretching modes

(approximated to Q=0), the Gaussian fitted values should be decreased by about 8.5 and 7.2 meV for NH_3 and ND_3 respectively, to account for the neutron recoil.

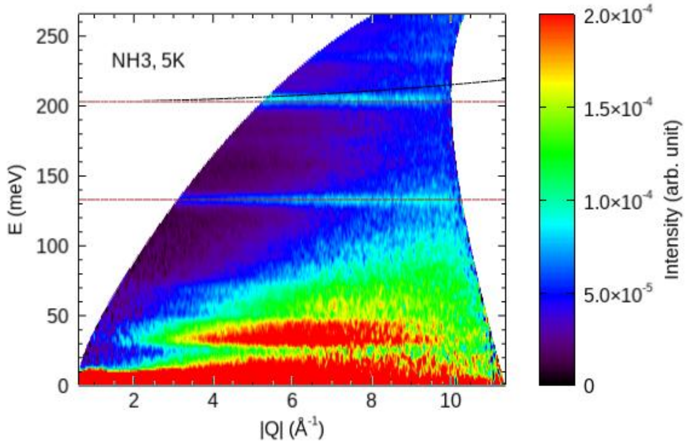

Figure S3. *S(Q,E)* spectrum for NH_3 measured with E_i=280 meV at 5 K, which shows that the intramolecular bending modes (at energies 133, 203 and 207 meV) are almost dispersionless. The black curve illustrates that the calculated shift for the mode at 203 meV in case of the neutron recoil scattering on free NH_3 molecule (M=17 a.u.) deviates from the observed almost flat behavior of the mode. Therefore, neutron recoil scattering on ammonia is insignificant for the data obtained with incident neutron energies E_i=280 meV and below.

Quantum Rotor Model

Excitation Energies (meV)
1.30×10^{-6}
1.30×10^{-6}
32.14
32.14
32.14

Table S1: First 5 energies in quantum rotor model. The first two energies correspond to the tunnel splitting, while the next three are to n=0→1 excitation in a quantum oscillator model

representing the excitation of the rotational vibration of NH_3. The latter three are what are measured in an INS experiment.

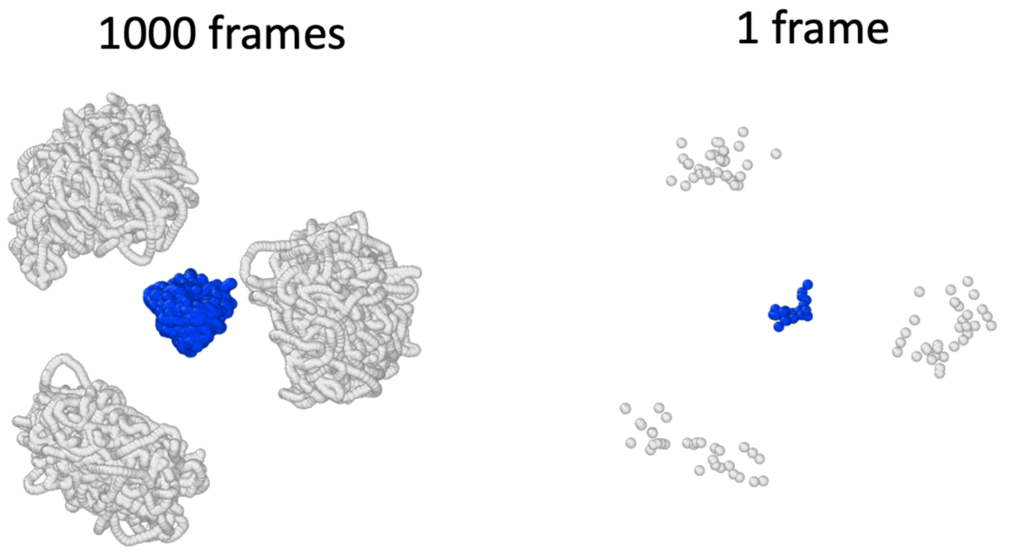

Figure S4. Beads of Ammonia Molecule in PIMD trajectory. Nitrogen colored in blue and hydrogen in white. Nitrogen and Hydrogen spheres are drawn to same size to better visualize effective phase space visited by both species.

Collective Excitations in Ammonia.

In liquid phase of ND_3 at 200K, coherent excitations can be seen resembling acoustic like phonons (similar to solid phase, see Fig. 1d-i) propagating from first sharp diffraction peak (Q~2.1Å^{-1}) in $S(Q,E)$, which is illustrated in figures S5a. Elastic line obtained from $S(Q,E)$ measured with SEQUOIA is illustrated in figure S6e. The 2D $S(Q,E)$ plot in figure S4a illustrates a splitting of the first sharp diffraction with increasing energy, resulting in a shift of the first peak to lower Q as energy increases. To better quantify this we plotted intensity along constant energy cuts, $S(Q,E\pm\Delta E)\times E$, with ΔE=0.5 meV for liquid deuterated ammonia ND_3 at T=200 K. As

S(Q,E)~1/E, we have presented the plot for *S(Q,E)×E*, so the energy cuts will be on the comparable intensity scale. This illustrated in figure S5b. The single peak at *Q*=2.1 A^{-1} for *E*=3.5 meV, split at higher energies, and the left peak moves to lower *Q* values, of 2 Å^{-1} for 6.5 meV, 1.8 Å^{-1} for 9.5 meV, 1.7 Å^{-1} for 12.5 meV, 1.5 Å^{-1} for 15.5 meV, and 1.2 Å^{-1} for 18.5 meV, which resemble acoustic like phonons propagation from the first sharp diffraction peak. This dispersion relation is plotted in figure S5c.

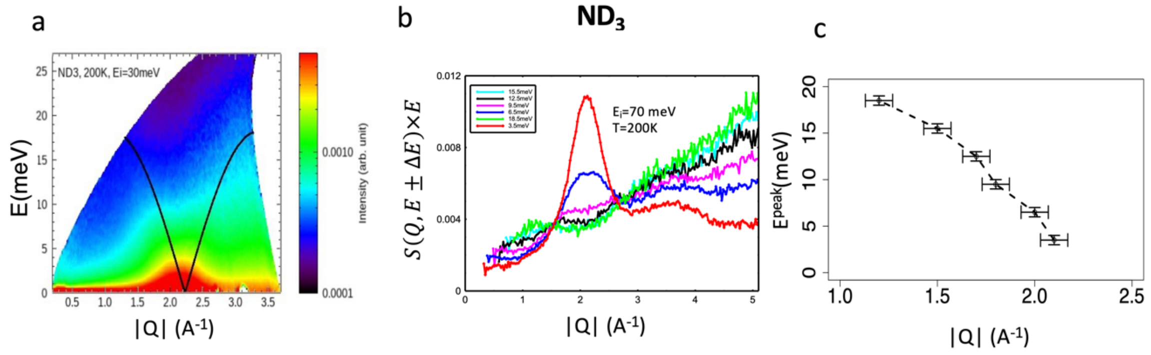

Figure S5. Low energy Dynamic Structure Factor for Liquid ND_3. (a) Show the Dynamic structure factor of ND_3 measured with SEQUOIA spectrometer for incident energy of 30 meV, with lines representing acoustic phonon like excitations. (b) Shows S(Q,E±ΔE)×E (ΔE=0.5 meV) for incident energy of 70 meV. With increasing energy, the first sharp diffraction peak splits, demonstrating behavior of acoustic like phonons propagating from the first sharp diffraction peak. (c) Shows dispersion like plot for lower energy peaks in (b) Error bars estimated for energy estimated as the energy window ΔE=0.5 meV, and for *Q* is estimated as ΔQ =0.14Å^{-1} which is twice the *Q* spacing resolution.

Density Functional Theory (DFT) Molecular Dynamics (MD) for Liquid ND_3

We performed DFT-MD simulations of liquid ND_3. Computation of the first sharp diffraction peak position (Fig S6a) is in good agreement with SEQUOIA measurement (Fig S6e). Computed radial distribution functions in Figs. S6b-d illustrate good agreement with previous neutron

experiments extracted from main-text Ref. 4,43, demonstrating the chosen functional describes structure of the liquid state well, especially the N-N peak which describes inter-molecular interactions.

To understand the N-H stretch hardening we examined the charge density overlap in solid and liquid phase. In comparison to liquid phase, we see smaller overlap of the charge density of neighboring molecules in the crystal phase. The threshold for charge density overlap in crystalline phase was found to be on contours of 0.013 e/Å^3, whereas in liquid phase molecules could be found sharing charge density iso-surfaces at almost twice the value seen in solid phase (see Fig. S7)

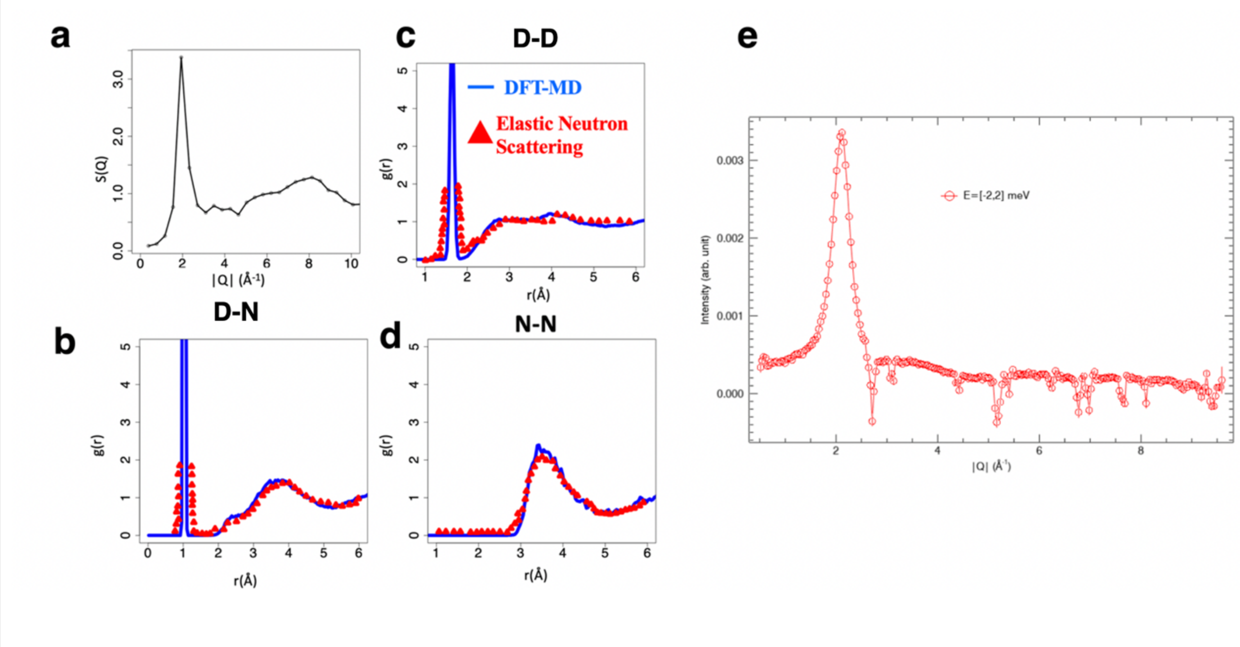

Figure S6. DFT-MD simulations of liquid deuterated ammonia structure. (a) Static structure factor computed for liquid ammonia. First sharp diffraction peak position is in good agreement with elastic line measurements for ND_3 on SEQUOIA (e). Negative intensity in (e) comes from self-shielding effect of subtracting empty aluminum container background. (b-d) Radial distribution functions of liquid ammonia computed from DFT-MD and those measured from neutron diffraction experiment extracted from main text refs 4,43.

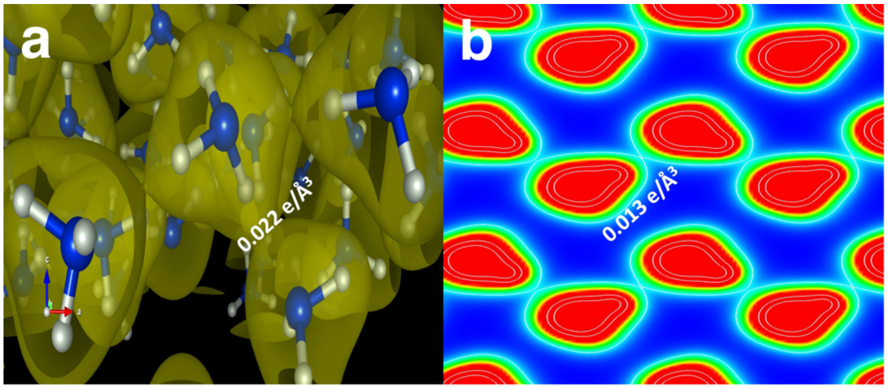

Figure S7. Charge density overlap in liquid and solid phases. In liquid phase (a), we plotted perspective view in liquid of ammonia molecules with large charge density overlap. In crystalline phase (b), we plotted [100] slice of charge density with iso-lines of constant density. Overlap is found at lines of 0.013 e/Å, nearly half the value at which ammonia molecules could share iso-surfaces of constant density in liquid phase.

Comparison of Optical and Neutron Data

Below table S2 compares optical data taken in ~~taken~~ main text references 2, 48, 49 for the solid phase to those measured by VISION and SEQUOIA for the inter-molecular modes and those taken by SEQUOIA for the intra-molecular modes for NH_3. A similar comparison in the liquid phase for the inter-molecular modes is given table S3.

	Optical 18K	Neutron 5K (SEQUOIA,VISION)
Acoustic	--	8.9, 8.75
Acoustic	--	10.9,10.65
Γ Translational	13.39	12.7, 12.4
M Translational	--	14.7,14.5
M Translational	--	16.0,15.5
Γ Translational	17.48	19.0,18.6

Γ Translational	17.48	19.0,18.6
Γ Translational	22.8	21.2,20.8
Rotational	--	30.4,29.75
Rotational	32.4	33.0,32.3
Rotational	37.13	38.4,39.5
Rotational	38.8	39.8,39.5
Rotational	44.72 & 45.47	44.5, 44.3
Rotational	--	55.2,54.0
Rotational	--	60.5,59.75
Rotational	66.08	67
Symm. Bend	131.05	133
Symm. Bend	132.97	133
Degen. Bend	202.83	203
Degen. Bend	204.57	207
Degen. Bend	208.04	
Symm. Stretch	397.18	Non-resolved 408.5
Degen. Anti-Symm. Stretch	417.82	Non-resolved 408.5
Degen. Anti-Symm. Stretch	418.8	

Table S2. Optical – neutron data comparison in solid phase.

	Optical (200K)	Neutron (200K)
Symm. Bend	132	132
Degen. Bend	203	205
Symm. Stretch	398	Non-resolved 412.5

Degen. Anti-Symm. Stretch	409	Non-resolved 412.5
Degen. Anti-Symm. Stretch	419	

Table S3. Optical – neutron data comparison in liquid phase.

Sample container for liquid ammonia

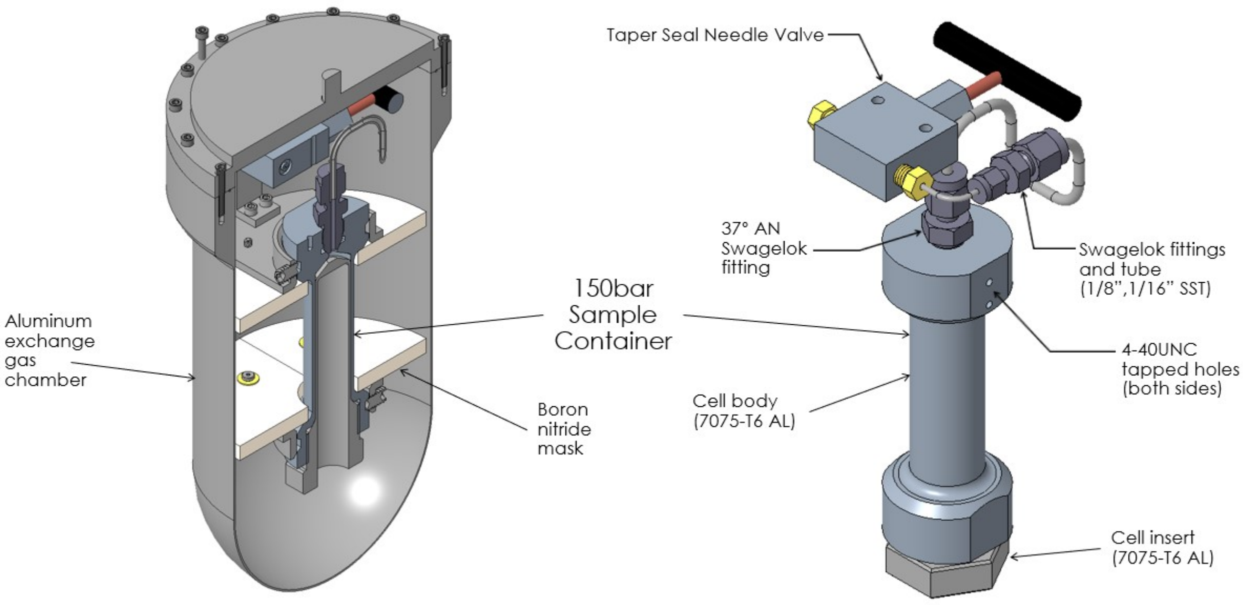

Figure S8. Sample container for liquid ammonia (center part), shielded by two horizontal BN-masks to prevent multiple neutron scattering. The radial gap for the NH_3 and ND_3 ammonia were 0.2 and 0.7 mm, respectively. The liquid ammonia was condensed in the container at T=77 K using a gas-handling system with calibrated volume of ammonia. After the ammonia was loaded, the valve of the container was closed, the container was heated to room temperature and placed inside larger thin-wall aluminum container in He glove-box, and the large container was

sealed with indium wire. The large container was attached to the cold-head of the bottom loading closed-cycle refrigerator, capable to provide base temperature 5 K. The temperature of the cold-head were regulated between 5 K and 300 K. The incoming neutron beam aperture in the experiment was 20 mm wide and 50 mm height. At room temperature ammonia in the container is in gas phase and the pressure can rise to 25 bar, therefore the container is designed to withstand a maximum pressure 150 bar (for safety reason).

Gaussian Fits

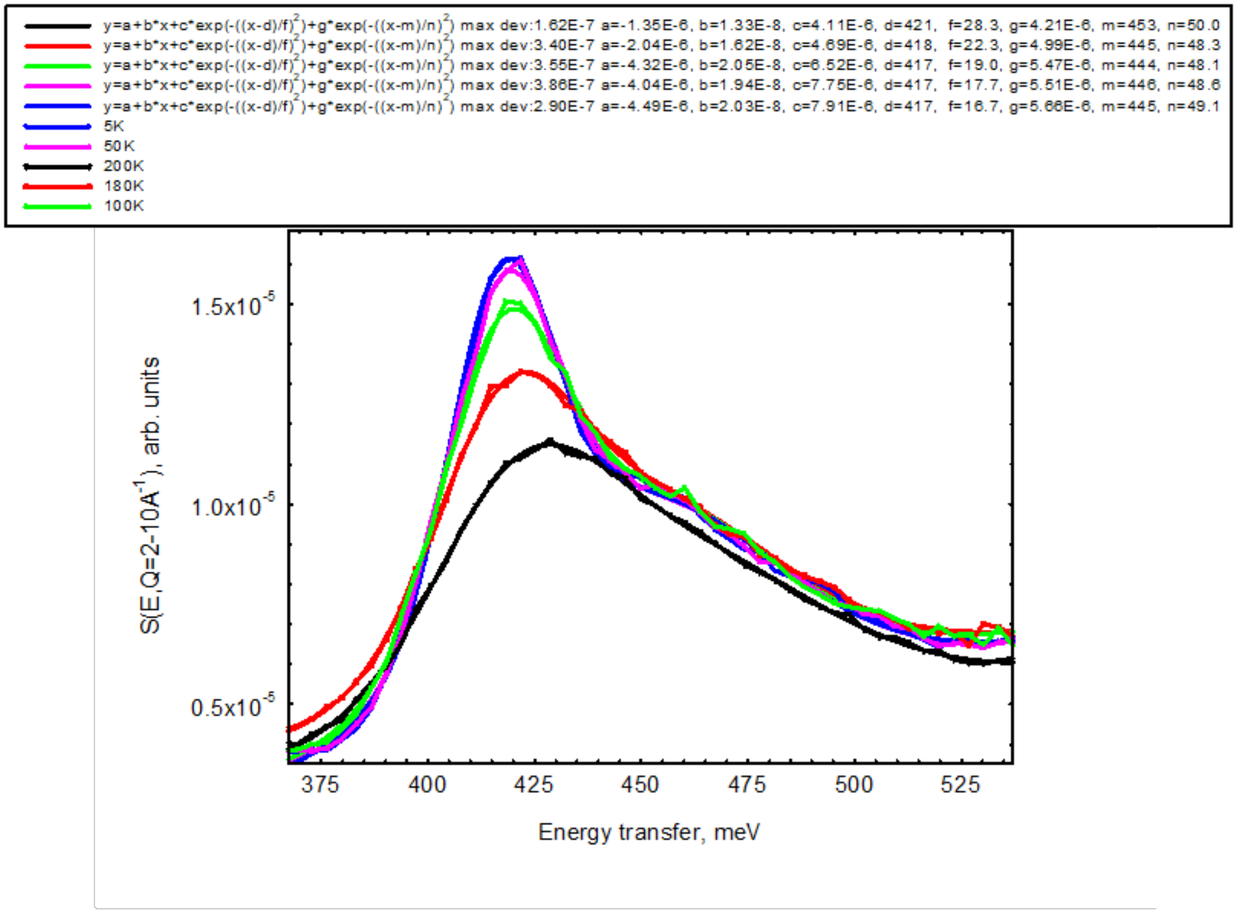

Figure S9. Gaussian fit to NH3 high energy stretching peak with linear +plus gaussian multi-phonon background.

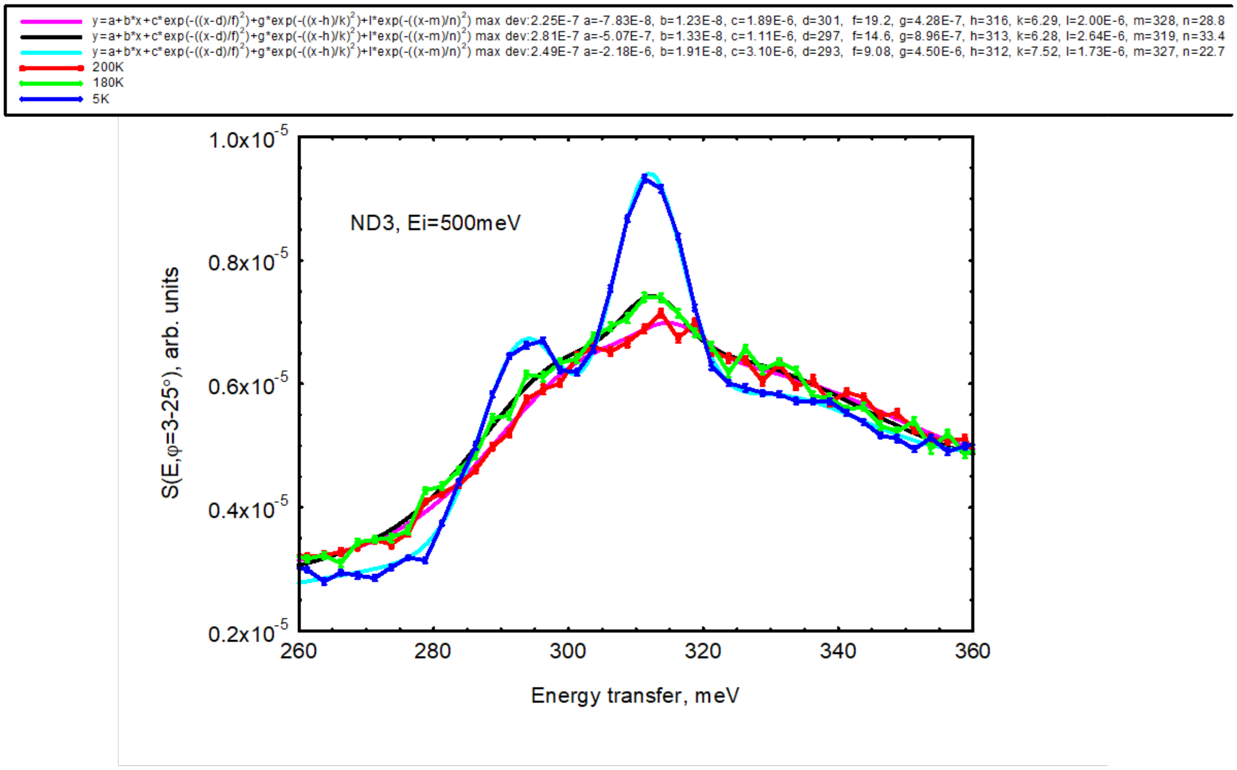

Figure S10. Double Gaussian fit to ND3 high energy stretching peaks with linear +plus gaussian multi-phonon background.
